# Comparison between Hydrofluoric Acid and Single-Component Primer as Conditioners on Resin Cement Adhesion to Lithium Silicate and Lithium Disilicate Glass Ceramics

**DOI:** 10.3390/ma14226776

**Published:** 2021-11-10

**Authors:** Alessandro Vichi, Riccardo Fabian Fonzar, Michele Carrabba, Chris Louca, Nicola Scotti, Claudia Mazzitelli, Lorenzo Breschi, Cecilia Goracci

**Affiliations:** 1 Dental Academy, University of Portsmouth, Portsmouth PO1 2QG, UK; chris.louca@port.ac.uk; 2 Private Practice, Studio Fonzar, 33030 Campoformido, Italy; riccardofabianfonzar@gmail.com; 3 Private Practice, Studio Associato, 53100 Siena, Italy; m.carrabba@yahoo.it; 4 Department of Surgical Sciences, Dental School Lingotto, University of Turin, 10126 Turin, Italy; nicola.scotti@unito.it; 5 DIBINEM, University of Bologna, 40125 Bologna, Italy; claudia.mazzitelli@unibo.it (C.M.); lorenzo.breschi@unibo.it (L.B.); 6 Department of Medical Biotechnologies, University of Siena, 53100 Siena, Italy; cecilia.goracci@unisi.it

**Keywords:** VITA Suprinity, e.max CAD, lithium disilicate, lithium silicate, hydrofluoric acid, silane, ceramic primer, Monobond

## Abstract

This study aimed at evaluating the effects of different surface conditionings on the microshear bond strength (µSBS) of a self-adhesive resin cement to VITA Suprinity (ZLS) and IPS e.max CAD (LD). Three surface conditioning protocols were performed on ZLS and LD before luting with a self-adhesive resin cement (RelyX Unicem 2, RXU): hydrofluoric acid (HF), HF + silane (HF + S), or Monobond Etch & Prime (EP). In each group, 15 cylindrical buildups of RXU were prepared on five milled bars and submitted to a µSBS test. Data were statistically analyzed with two-way ANOVA and Tukey’s post hoc test (*p* < 0.05). Failure modes were recorded and classified as adhesive, mixed, cohesive in resin, or ceramic, and statistically analyzed with Fisher’s exact test (*p* = 0.05). One additional bar per group was used for the morphological characterization of the conditioned surface by means of SEM. The material per se did not significantly influence adhesion (*p* = 0.744). Conditioning protocol was a significant factor: EP yielded significantly higher μSBS than HF (*p* = 0.005), while no significant differences emerged between EP and HF + S (*p* = 0.107), or HF + S and HF (*p* = 0.387). The material-conditioning protocol interaction was not statistically significant (*p* = 0.109). Significant intergroup differences were found in distribution of failure modes: mixed failures were predominant in the ZLS/EP group, while the other groups showed a prevalence of adhesive failures. The self-etching primer showed promising results in terms of immediate bond strength of a self-adhesive resin cement to lithium-silica-based glass ceramics, suggesting its alternative use to hydrofluoric acid and silane conditioning protocols.

## 1. Introduction

Lithium-silica-based glass ceramics represent a viable alternative to conventional porcelain fused to metal to fabricate monolithic single-unit restorations with high-level mechanical properties and a toothlike appearance [1]. Heat-pressed ingots and CAD/CAM blanks are available for material processing. Among the blanks, the conventional lithium disilicate and the more recent zirconia-reinforced lithium silicate were developed for the CEREC system, but they can now be used in several CAD/CAM systems with different notches or adapters [2,3]. After the digital wax-up, the monolithic restoration is milled, crystallized, finished, and finally cemented with nonadhesive or adhesive techniques [4,5,6], although the nonadhesive option is progressively being abandoned. Resin cements are commonly used to bond the restorations to the tooth [7,8]. To simplify the clinical procedure, self-adhesive resin cements are utilized to chemically adhere to the abutment without any dental surface pre-treatment [9,10,11,12].

Bonding to glass ceramics requires the intaglio surface of the restoration to be mechanically and/or chemically treated before cementation [5,6,7,8,9,10,11,12,13]. Different chemo/mechanical surface conditionings have been proposed over time to promote microretentions; these include sandblasting, Er:YAG laser ablation, and acid etching [5,7,8,13,14,15,16,17,18,19,20,21,22,23,24,25,26]. Among the available surface pretreatments, hydrofluoric acid is widely recommended for effective conditioning of lithium-silica-based glass ceramics [5,8,14,17,18]. After etching, a dissolution of the glassy matrix occurs, and the surface becomes rough [19], promoting mechanical interlocks between resin cements and crystals [5,8,13,15,19]. To maximize the affinity with polymers, silane agents are applied on the etched surface, enhancing the physio-chemical interaction between the luting agent and the glass ceramic [16,18,20,21,22,23].

Although hydrofluoric acid etching solutions are considered effective in promoting resin cement adhesion to glass ceramics, their use has been prohibited in some countries because of their toxicity [24]. Alternatively, a new self-etching ceramic primer with lower toxicity, based on ammonium polyfluoride and trimethoxypropyl methacrylate, was proposed to simultaneously etch and prime the intaglio surface [25]. Its effectiveness on lithium disilicate glass ceramics is still debated in the literature and, at present, no data have been published on its efficacy on lithium silicate zirconia-reinforced glass ceramics.

Therefore, the objective of the present study was to evaluate the microshear bond strength of a self-adhesive dual-cure resin cement to VITA Suprinity (ZLS) and IPS e.max CAD (LD), after conditioning with a hydrofluoric acid solution (HF), followed by the application of a silane agent (HF + S), or with the use of a self-etching ceramic primer (EP). The tested null hypothesis was that no statistically significant difference existed in the bond strength (µSBS) achieved by the cement to VITA Suprinity and IPS e.max CAD, following the three compared conditioning protocols.

## 2. Materials and Methods

### 2.1. Microshear Bond Strength Test (µSBS)

CEREC CAD/CAM precrystallized blocks of zirconia-reinforced lithium silicate (VITA Suprinity, VITA Zahnfabrik, Bad Säckingen, Germany, ZLS) and lithium disilicate (IPS e.max CAD, Ivoclar Vivadent, Schaan, Liechtenstein, LD) were used for the study (n = 6 blocks per material). Each block was milled (CEREC inLab MC XL, Sirona, Bensheim, Germany) to obtain bars of 4.0 ± 0.2 mm in width, 1.2 ± 0.2 mm in thickness, and 15 ± 0.2 mm in length. The milled bars were placed in their proprietary firing tray, isolated with firing paste, and a final crystallization was performed in the respectively recommended furnaces: VITA Vacumat 6000 (VITA Zahnfabrik, Bad Sackingen, Germany) for ZLS and EP 600 Combi (Ivoclar Vivadent, Schaan, Liechtenstein) for LD, according to the manufacturer’s instructions.

The bars were then randomly divided into 3 equally sized groups for each material. Groups were defined depending on the protocol used for conditioning: HF, HF + S and EP. For each group, 5 bars were preliminarily cleansed by an ultrasonic bath in demineralized water for 3 min and dried with an oil-free air stream. To test the materials on a standardized bonding area, an aluminum split mold was used to hold onto the bar a 0.5 mm thick plastic-wax mold that exhibited 3 equally spaced holes (internal diameter: 0.8 mm). Each bar thus provided 3 bonding areas for testing [26].

In the HF group, a 4.9% hydrofluoric acid solution (VITA Ceramics Etch, VITA Zahnfabrik, Bad Sackingen, Germany for ZLS; IPS Ceramic Etching Gel, Ivoclar Vivadent, Schaan, Liechtenstein, for LD), was applied for 20 s with a syringe and scrubbed with a brush on the bonding area. The bonding surface was rinsed for 15 s with an air-water spray and dried with an oil-free air stream.

In the HF + S group, HF etching was followed by the application of a silane solution (S). The ethanol-based silane coupling solution (RelyX Ceramic Primer, 3M Espe, St. Paul, MN, USA) was applied on the etched surface with a syringe, scrubbed with a brush, and left in place for 60 s. Then, the bonding surface was dried with an oil-free air stream until the solvent was completely evaporated.

In the EP group, the self-etching primer (Monobond Etch & Prime, Ivoclar Vivadent, Scheen, Liechtenstein) was applied on the bonding area with a syringe, scrubbed for 20 s with a brush, and left in place for 40 s. The bonding surface was rinsed with air-water spray for 15 s and dried with an oil-free air stream.

The self-adhesive dual-cure resin cement (RelyX Unicem 2, 3M ESPE, ST. Paul, MN, USA) was applied onto the bonding surface up to the upper limit of the plastic mold (0.5 mm). The material was cured for 40 s using an LED light-curing unit (Elipar DeepCure-S; 3M ESPE, St. Paul, MN, USA) with a wavelength of 430–480 nm and output of 1.470 mW/cm^2^. Fifteen specimens (n = 15) were obtained for each group and examined using magnifying loupes (4.5×) to verify that the buildup did not exhibit defects. Then, specimens were secured in a vice and attached to a shear-testing jig. A stainless-steel wire (0.2 mm diameter) was looped around the cement buildup and kept close to the bonded substrate. Through a universal testing machine (Triax 50, Controls, Milan, Italy) at a crosshead speed of 1 mm/min, the wire loop applied a shear force parallel to the adhesive interface until bond failure [26]. The microshear bond strength (µSBS) was calculated in MPa by dividing the load at failure by the surface area (mm^2^) of each specimen. The failure mode of the debonded specimens was determined using a stereomicroscope (SMZ 800, Nikon, Tokyo, Japan) at 120× magnification and was classified as adhesive, mixed, cohesive in the resin cement, or cohesive in the ceramic.

### 2.2. Statistical Analysis

Having preliminarily verified that µSBS data met the requirements of normal distribution (Shapiro–Wilk test) and homogeneous group variances (Levene’s test), the two-way analysis of variance (ANOVA) was used with bond strength data as the dependent variable, with material and conditioning protocol as factors. Tukey’s test was applied for post hoc comparisons as needed.

The between-group differences in the distribution of failure modes were statistically analyzed using the Fisher’s exact test, followed by a series of Fisher’s exact tests with Bonferroni’s correction for post hoc comparisons. In all the statistical tests, the level of significance was set at α = 0.05. The statistical analysis was processed by SigmaPlot 11.0 (Systat Software, Inc., San Jose, CA, USA) software.

### 2.3. Scanning Electron Microscope Evaluation (SEM)

For each experimental group, 1 additional bar was used for SEM observation of the conditioned surface. Prior to conditioning, specimens were ultrasonically cleansed in demineralized water for 3 min and dried with an oil-free air stream. The same conditioning modalities as in the preparation of specimens for µSBS test were then followed. Bars were mounted on stubs and gold sputter-coated (SC7620, Quorum, Laughton, U.K.). The specimens were observed with SEM (JSM-6060L, JEOL, Tokyo, Japan) at 5000× magnification.

## 3. Results

### 3.1. Microshear Bond Strength Test (µSBS)

Descriptive statistics of µSBS data and statistically significant results are reported in Table 1. Regardless of the conditioning protocol, no statistically significant difference emerged in the adhesion to ZLS and LD (*p* = 0.744). The conditioning protocol was found to be an influential factor (*p* = 0.005) with significantly superior bond strengths measured after the application of EP than after conditioning with HF. No significant differences were recorded in the bond strengths comparison between HF and HF + S (*p* = 0.387), or between HF + S and EP (*p* = 0.107). The interaction between material-conditioning protocols was not statistically significant (*p* = 0.109).

The distribution of failure modes is reported in Table 2. The Fisher’s exact test revealed the existence of a statistically significant difference in the distribution of failure modes among the groups (*p* < 0.001). The post hoc comparisons highlighted that when ZLS was treated with EP, mixed failures were significantly more frequent than adhesive failures. In all the other groups, a prevalence of adhesive failures was observed.

### 3.2. Scanning Electron Microscope Evaluation (SEM)

**Figure 1:**
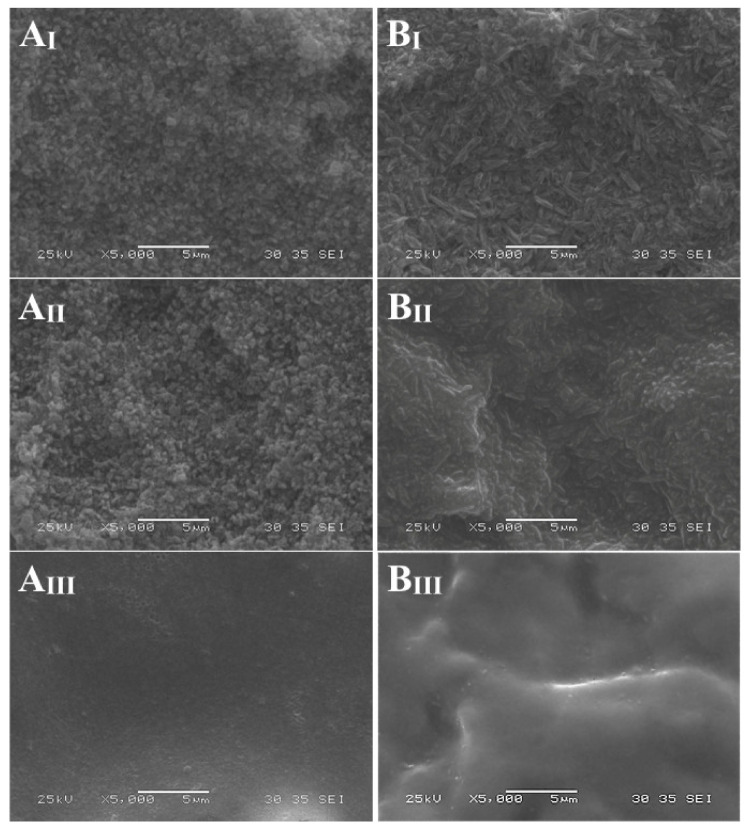
VITA Suprinity (**A**) and IPS e.max CAD (**B**) after conditioning with hydrofluoric acid (**I**), hydrofluoric acid and silane (**II**), Monobond Etch & Prime (**III**). SEM images at 5000×.

By conditioning with HF gel, the glassy phase superficially dissolved, and the crystalline phase became visible. ZLS exhibited shorter, densely distributed, round crystals, as opposed to the more elongated club-like crystalline microstructure of LD. The etching pattern was less evident after the application of S, particularly for LD. No morphological changes were appreciable on the surface of ZLS and LD after conditioning with EP (Figure 1).

## 4. Discussion

The present investigation evaluated different procedures for bonding to VITA Suprinity and IPS e.max CAD. The result that conditioning protocol had a significant influence on resin cement adhesion, while the material was not an influential factor, led to partial rejection of the formulated null hypothesis.

HF etching is the traditional treatment for intaglio surface conditioning. By HF etching, the glassy and second crystalline phases dissolve and the crystal network is exposed [27,28]. The contact area extends [29], while roughness and wettability of the ceramic surface increase [8,17,29]. Resin cement can therefore infiltrate the crystalline microstructure and, once polymerized, be mechanically retained [30].

Even if the micromechanical retention of the cement into the etched surface is considered more important than the chemical bond to the inorganic phase of the glass ceramics, a further improvement in the bonding to lithium-silica-based glass ceramics can be obtained by applying silane after HF etching [4,8,19,22,29,31,32,33,34,35]. In the present investigation, a methacrylate propyl trimethoxysilyl silane was used in one of the experimental groups after HF conditioning.

The similar results obtained for HF and HF + S in the present study might question the usefulness of applying silane after HF etching. While the contribution of silanization to adhesion to glass ceramics was reported to be statistically significant, its clinical relevance has not yet been proved.

Although HF etching is considered the gold standard conditioning method for bonding to glass ceramics’ intaglio surfaces, HF is toxic even in the dissolute state. After application it shortly reaches the deeper tissue layers, releasing freely dissociable fluoride anions. Fluoride is toxic as well due to its high reactivity with cellular Ca^+^ and Mg to form insoluble salts, resulting in cellular death and necrosis. Moreover, HF can burn the skin and penetrate through the epidermis, dermis, and subcutaneous tissues, causing severe destruction to the underlaying bone. Even though the extent of these injuries is related to HF concentration, its use as a ceramic etching gel is banned in some countries.

EP conditioner was developed to overcome this toxicity hazard. EP is a newly introduced one-bottle system combining ammonium polyfluoride for etching and priming and trimethoxypropyl methacrylate for silanization. Ammonium polyfluoride is a weak acid salt and an intermediate to the production of HF [36].

The application of a diluted HF solution dissolves silicon ions in the glassy state. Silicon and fluoride show high affinity, resulting in soluble silicon-fluoride derivates that can be easily rinsed with water. This system is intended to simultaneously etch and prime the glass ceramics, thus reducing clinical time.

The mean μSBS values measured in the present study were 24.66, 23.13, and 28.42 MPa after conditioning ZLS with HF, HF + S, and EP, respectively. LD yielded 19.73, 27.24, and 31.06 MPa with the same protocols, respectively. A direct comparison with bond strength data reported in other studies is quite complex because a wide range of values can be found in the literature for lithium (di)silicate ceramics, ranging from 10.1 to 45.7 MPa [4,8,9,15,22,30,32,33,34,35,37,38,39,40,41,42,43,44]. This wide range might be explained by different testing setups. For the present study, the µSBS test used allowed generating stresses parallel to the bonding interface, more truthfully reproducing the clinical situation when the luted crown undergoes displacement forces [45]. In addition, as opposed to the microtensile bond strength test, no pretest failures were expected to occur since specimens were not subjected to trimming after bonding [32,33,34,35,36,37,38,39,40,41,42,43,44,46]. An intrinsic limitation of the μSBS design is the lack of seating pressure during cement application [47]. Due to the absence of hydraulic pressure, the resin cement might have had limited penetration into the milling grooves, possibly accounting for the finding of lower mean bond strengths than those recorded with other testing methodologies.

A peculiarity of the present study is that, different from most of the published papers in which the efficacy of the bonding protocol was tested on standard smoothed surfaces, here, ZLS and LD underwent the conditioning process after milling, as it occurs clinically. Smoothing the surfaces with silicon carbide papers (SiC) and diamond pastes prior to conditioning, as reported in previous studies [19,29,31,33,37,48], removes any preexisting mechanical retention. Such a method does not faithfully replicate the in vivo situation, where conditioners are applied on the post-milling coarse surface [44]. In the present study CAD/CAM modeling was used purposely to directly mill the substrate bars. Only Prado et al. [49] used a similar approach, although these authors utilized the residual part of the block after milling, which is usually rougher than the milled surfaces.

The comparison between the tested protocols showed stronger adhesion under conditioning with EP than with HF. However, no differences were found when comparing the bonding efficacy of HF with HF + S and HF + S compared with EP.

In the literature, conclusive evidence in the comparison between HF + S and EP has not yet been provided. In this study, no significant difference emerged between HF + S and EP. This finding is in line with the results of Román-Rodríguez et al. [38]. The authors reported similar shear bond strengths on LD after pretreatments with HF + S (26.53 MPa) and EP (23.52 MPa). Additionally, Maier et al. [50] in a tensile SBS test study on LD found that HF + S and EP had higher values than HF and no treatment. Similarly, Lyann et al. [51] reported that in a tensile SBS study on LD using three different cements, no differences existed between HF + S and EP, which yielded higher strengths than phosphoric acid + S, S alone, and no treatment. On the other hand, El-Damanhoury and Gaintantzopoulou [37] found in a SBS study that HF + S (37.60 MPa) was more effective than EP (28.06 MPa) in promoting adhesion to LD. Likewise, Prado et al. [49] reported for LD a higher mean μSBS with HF + S than with EP. A similar outcome was obtained by Lopes et al. [52]. These authors assessed by means of a μSBS test the effect on LD of several different market formulations of HF followed by silane and EP, and found that the latter achieved the lowest bond strength. Dönmez [53] also recorded lower SBS on LD with EP than with HF etching either for 20 or 60 s, without silanization. Such conflicting results do not allow, at present, a definitive conclusion on this issue. No other data than the ones collected in the present study are available in the literature for the effect of EP on VITA Suprinity intaglio surfaces.

While the material did not significantly influence bond strength, failure mode distribution was different between the two ceramics. ZLS showed more mixed failures than adhesive failures compared to LD. Differences in material composition and mechanical properties might account for this outcome. Zirconium oxide content differs between ZLS (8.0–12.0) and LD (0.0–8.0). Through the functional phosphate monomers, RelyX Unicem 2 can chemically bond to zirconium oxides and, consequently, to ZrO_2_-containing glass ceramics [35]. The greater zirconia content within ZLS may account for the higher prevalence of mixed failures observed for this ceramic [26]. Concerning the influence of the conditioning protocol on failure mode, the outcome that mixed fractures were more frequent than adhesive failures after conditioning with EP corresponded well with the finding of higher μSBS on EP-treated specimens [37,38].

When the effect of the conditioning protocol was observed under SEM, EP showed a less pronounced etching pattern than HF and HF + S on both LD and ZLS (Figure 1). When treated with HF and HF + S, ZLS showed a smaller crystal phase compared to LD, typical of the materials. Etching with EP determined a less defined crystal phase compared to HF and HF + S groups, which showed a higher degree of demineralization. These observations have been generally confirmed in several studies [38,49,51,52,53,54].

On both prosthetic substrates, the new conditioning primer EP performed significantly better than HF and comparably to HF + S. Therefore, as a general indication, the choice between the EP or HF + S protocol should be based on other factors than bond strength, such as ease of use, timing of the procedure, and hazard concerns. In all these aspects, EP presents as a preferable solution to HF + S.

In this in vitro investigation, EP emerged as a convenient alternative for glass ceramic conditioning. Nevertheless, for a more thorough evaluation, the two procedures should also be compared in vivo, assessing the long-term bonding at the ceramic–resin cement interface.

## Figures and Tables

**Table 1:** Mean µSBS (MPa) and standard deviations (SD) of RelyX Unicem 2 to VITA Suprinity (ZLS) and IPS e.max CAD (LD) after conditioning with hydrofluoric acid (HF), hydrofluoric acid plus silane (HF + S), and Monobond Etch & Prime (EP). Different letters label statistically significant differences in bond strength.

Material	Conditioning Protocol	Mean	SD	N
ZLS 25.51 (8.94)	HF	24.66	11.02	15
	HF + S	23.13	8.97	15
	EP	28.42	5.68	15
LD 26.23 (10.53)	HF	19.73	8.38	15
	HF + S	27.24	12.96	15
	EP	31.06	6.41	15
Conditioning Protocol				
HF ^b^ 22.19 (9.94) HF + S ^ab^ 25.12 (11.11) EP ^a^ 29.81 (6.13)				

**Table 2:** Failure mode: M = mixed, A = adhesive, CR = cohesive in resin cement, and CC = cohesive in ceramic. Different letters label statistically significant differences in failure modes’ distribution.

		Groups					
		ZLS HF ^a^	ZLS HF + S ^a^	ZLS EP ^b^	LD HF ^a^	LD HF + S ^a^	LD EP ^a^
**Failure modes**	M	4	4	14	3	0	0
	A	11	11	1	12	13	13
	CR	0	0	0	0	2	2
	CC	0	0	0	0	0	0

## Data Availability

The data presented in this study are available on request from the corresponding author. The data are not publicly available due to University policy on access.
